# Amoxicillin plus temocillin as an alternative empiric therapy for the treatment of severe hospital-acquired pneumonia: results from a retrospective audit

**DOI:** 10.1007/s10096-015-2406-x

**Published:** 2015-05-19

**Authors:** H. Habayeb, B. Sajin, K. Patel, C. Grundy, A. Al-Dujaili, S. Van de Velde

**Affiliations:** Ashford & St. Peter’s Hospitals NHS Foundation Trust, Guildford Road, Chertsey, KT16 0PZ Surrey UK; Royal Berkshire Hospital NHS Foundation Trust, Craven Road, RG1 5AN Reading, UK; Eumedica s.a., Chemin de Nauwelette 1, 7170 Manage, Belgium

## Abstract

A formulary decision was made at a large provider of acute hospital services in Surrey to replace piperacillin/tazobactam with amoxicillin+temocillin for the empiric treatment of severe hospital-acquired pneumonia. This decision was made because the use of broad-spectrum-β-lactam antibiotics is a known risk factor for *Clostridium difficile* infection (CDI) and for the selection of resistance. After the antibiotic formulary was changed, a retrospective audit was conducted to assess the effect of this change. Data from patients hospitalised between January 2011 and July 2012 for severe hospital-acquired pneumonia and treated empirically with piperacillin/tazobactam or amoxicillin+temocillin were reviewed retrospectively. Clinical characteristics of patients, data related to the episode of pneumonia, clinical success and incidence of significant diarrhoea and CDI were analysed. One hundred ninety-two episodes of severe hospital-acquired pneumonia in 188 patients were identified from hospital records. Ninety-eight patients received piperacillin/tazobactam and 94 amoxicillin+temocillin. At baseline, the two treatment groups were comparable, except that more patients with renal insufficiency were treated with piperacillin/tazobactam. Clinical success was comparable (80 versus 82 %; *P* = 0.86), but differences were observed between piperacillin/tazobactam and amoxicillin+temocillin for the rates of significant diarrhoea (34 versus 4 %, respectively; *P* < 0.0001) and for CDI (7 versus 0 %, respectively; *P* < 0.0028). This preliminary study suggests that the combination amoxicillin+temocillin is a viable alternative to piperacillin/tazobactam for the treatment of severe hospital-acquired pneumonia. This combination appears to be associated with fewer gastrointestinal adverse events. Further studies are needed to evaluate the place of amoxicillin+temocillin as empiric treatment of severe hospital-acquired pneumonia.

## Introduction

The widespread use of broad spectrum antibiotics has led to the emergence of multidrug-resistant bacteria, especially multidrug-resistant Gram-negative bacilli [1–3]. Since the future of all antibiotics is threatened by the spread of multidrug-resistant (MDR) bacteria, it is important to decrease their global consumption and to favour narrow spectrum antibiotics whenever possible [4, 5].

Piperacillin/tazobactam is often used as first-line treatment for several types of severe infection including: severe hospital-acquired pneumonia (HAP), septicaemia/sepsis, urosepsis, severe cholecystitis/cholangitis and neutropenic sepsis. To limit the use of this broad-spectrum antibiotic, a formulary decision was made at a large provider of acute hospital services in Surrey (serving a population of 380,000 people) to replace piperacillin/tazobactam with an original antibiotic combination (amoxicillin plus temocillin) for the empiric treatment of severe hospital-acquired pneumonia. This combination covers *Streptococcus pneumoniae*, Enterobacteriaceae (including extended-spectrum β-lactamase (ESBL)-producing organisms) and *Haemophilus influenzae*, but not *Pseudomonas aeruginosa* nor *Staphylococcus aureus*. For this reason, the hospital formulary was updated to cover the following situations: (i) teicoplanin instead of amoxicillin if methicillin-resistant *Staphylococcus aureus* was suspected, (ii) add flucloxacillin to amoxicillin plus temocillin for methicillin-sensitive *S. aureus* or (iii) add metronidazole if aspiration pneumonia was thought likely.

Temocillin is a narrow spectrum antibiotic active against Gram negative bacteria and more particularly against the Enterobacteriaceae and *Haemophilus influenzae,* while non-fermenters, Gram-positive aerobes and strict anaerobes are not included in its spectrum [6]. Temocillin has been marketed since 1984 in Belgium, Luxembourg and since 1989 in the United Kingdom. In December 2014, it has been approved in France in response to the rise in ESBL-producing coliforms. In vitro and clinical data suggest temocillin is a potential alternative to carbapenems because of its stability against most β-lactamases including ESBLs, AmpC enzymes and even some carbapenemases [7–10].

After the antibiotic formulary was changed, a retrospective audit was conducted to assess the effect of this change.

## Materials and methods

This audit was a service evaluation and was conducted for internal hospital guidance. It was initiated at the request of the Infection Control Committee and the Antibiotic Group in Ashford & St. Peter’s Hospitals NHS Foundation Trust. According to the UK national research ethics service, neither consent, nor ethical approval was needed as the data collection was retrospective and for local guidance, not a formal research project.

A sample size calculation was performed based on a non-inferiority methodology. A minimum of 92 episodes per group would be needed to detect a difference of 15 % between groups based on the following assumptions: alpha of 5 %, beta of 90 % and clinical cure rate set at 86 % in each group.

The formulary decision replacing piperacillin/tazobactam with amoxicillin plus temocillin took place in November 2011. From our clinical coding department, 247 episodes of severe HAP were retrieved. Those treated with piperacillin/tazobactam were collected from January 2011 to November 2011 and those treated with amoxicillin plus temocillin from November 2011 to July 2012.

Medical notes of patients with HAP were then retrieved and reviewed. The diagnosis of severe HAP was confirmed if all of the following factors were present: onset ≥ 48 h after hospitalisation, new/persistent otherwise unexplained infiltrates on chest X-ray, increased oxygen requirement, temperature <36 or >38.4 °C, CURB-65 score >2. Although the CURB-65 score has been validated for community-acquired pneumonia (CAP) [11] and not HAP, it may help clinicians to differentiate between severe HAP and mild HAP.

In accordance with local guidelines, daily doses of antibiotics were: 4 g/0.5 g piperacillin/tazobactam three times daily; 1 g amoxicillin three times daily and 2 g temocillin twice daily [or 1 g twice daily if creatinine clearance (CrCl) 30–60 mL/min or 1 g once daily if CrCl 10–30 mL/min]. Patients treated empirically with piperacillin/tazobactam or amoxicillin plus temocillin for at least 3 days were included in the present analysis. Patients with penicillin allergy or those who received a concomitant antibiotic not included in local guidelines were excluded. Patients having ventilated-acquired pneumonia (VAP) were also excluded as empiric treatment remains piperacillin/tazobactam.

Patient demographics, creatinine clearance (CrCl), lung injury prediction score, co-morbidities, microbiological data, prior and concomitant antibiotics, hospitalisation time, clinical outcome and in-hospital mortality were collected. Clinical outcome was assessed on day 3, 5 and 7 and validated by two blinded reviewers based on the analysis of the medical notes, clinician documentation of the chest X-ray findings and its progress, patient’s clinical progress and clinical observations (e.g., oxygen saturation, respiratory rate, and temperature) and infection markers from blood pathology results. Clinical outcomes were recorded as cure (improved clinical condition on day 5), failure (worsening of the clinical condition), relapse (recurrence of signs of pneumonia after day 7 among those who were cured) or indeterminate (inability to decide on the clinical outcome on day 3). Unless the assessment of efficacy was unclear, each reviewer validated different cases.

Incidence of significant diarrhoea (at least two episodes of type 6 or 7 stools on Bristol stool chart in 24 h) and CDI cases were recorded. For the patients included prior to 2012, the diagnosis of CDI relied on the detection of toxins A and B in faeces. As the sensitivity of these tests has been questioned in the literature [12], a two-step process (glutamate dehydrogenase and toxin immunoassay) is now in place in the hospital.

Statistical analyses were performed using JMP 10 (SAS Institute). Continuous and nominal variables were compared using Student's *t*-test and Fisher’s exact test, respectively. Each variable that was statistically significant on univariate analysis was entered into a multivariate model. Effect likelihood ratio tests after nominal logistic fit were performed for multivariate analysis.

## Results

### Demographic, baseline clinical data

One hundred ninety-two episodes of severe HAP in 188 patients met the inclusion criteria. Ninety-four episodes of HAP in 92 patients were treated with piperacillin/tazobactam and 98 episodes in 96 patients with amoxicillin plus temocillin. Clinical characteristics of patients are shown in Table 1. The two treatment groups were comparable in terms of demographics and severity of disease. However, more patients with renal insufficiency were treated with piperacillin/tazobactam.

**Table 1:** Characteristics of patients and infections

Characteristics	Piperacillin/tazobactam	Amoxicillin+temocillin	*P*-value
Patients			
Number of patients	92	96	NS
Male (%)	36 (39 %)	47 (49 %)	NS
Age (year)^a^	79 ± 13	80 ± 14	NS
Patients with renal insufficiency (%)^b^	49	31	<0.05
Charlson’s comorbidity index	5.9 ± 2.4	6.0 ± 2.5	NS
Hospitalised in intensive care	18 (20 %)	18 (19 %)	NS
Episodes of hospital-acquired pneumonia (HAP)			
Number of episodes	94	98	NS
LIPS score^a^	5 ± 2.0	5 ± 1.8	NS
ARDS (n)	16	14	NS
Length of hospital stay (days)^a^	30 ± 21	29 ± 18	NS
Duration of antibiotic treatment (days)^a^	6.7 ± 1.6	6.8 ± 1.5	NS
Previous antibiotic treatment (%)^c^	50	47	NS

*NS* not significant, *LIPS* lung injury prediction score, *ARDS* acute respiratory distress syndrome

^a^ Mean ± standard deviation

^b^Creatinine clearance < 60 mL/min

^c^Defined as the administration of any antibiotic prior to the administration of piperacillin/tazobactam or amoxicillin plus temocillin during the same hospital stay as the episode of HAP

About a fifth of the patients (36/188) were hospitalised in the intensive care unit (ICU), and 30/36 developed acute respiratory distress syndrome (ARDS). All patients who developed ARDS had a lung injury prediction score (LIPS) score of ≥7 and all patients with a LIPS score of ≥8 developed ARDS.

### Characteristics of HAP episodes and antibiotic treatment

Identification of a bacterial pathogen was not reported in 85 % of cases. Two patients in the piperacillin/tazobactam group had *P. aeruginosa* in their sputum compared to none in the amoxicillin plus temocillin group. No patients in either group had positive blood cultures for *P. aeruginosa*. One patient in the piperacillin/tazobactam group had a positive sputum culture for *S. aureus* compared to no patients in the amoxicillin plus temocillin group. No patients in either group had a positive blood culture for *S. aureus*.

Patients were treated according to the local guidelines. Three episodes in the piperacillin/tazobactam group received concomitant teicoplanin and one episode also received metronidazole. In ten episodes in the amoxicillin plus temocillin group patients received concomitant metronidazole. Table 1 shows the duration of antibiotic therapy and length of hospital stay, which were comparable between the two groups.

### Clinical outcome and in-hospital mortality

**Fig. 1:**
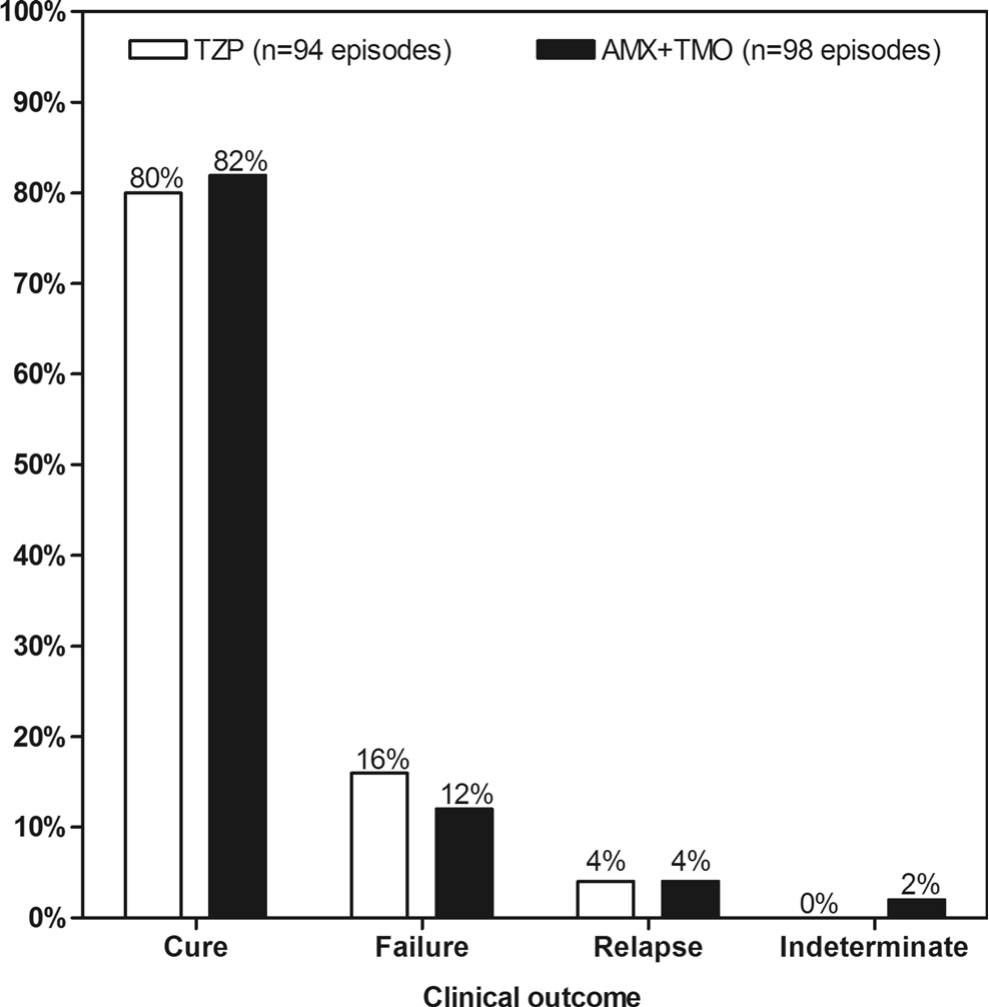
Clinical efficacy of treatment groups. Comparison of clinical outcomes between piperacillin/tazobactam (TZP) [*white bar*] and amoxicillin plus temocillin (AMX+TMO) [*black bar*] groups. No significant difference was observed

Clinical cure rate was 80 % [75/94] for piperacillin/tazobactam and 82 % [80/98] for amoxicillin plus temocillin, whilst clinical failure occurred in 15 and 12 episodes, respectively (Fig. 1). Four relapses occurred in both of the study groups, and two episodes were deemed indeterminate in the amoxicillin plus temocillin group.

On univariate analysis, treatment duration (*P* < 0.0001), the presence of renal insufficiency (*P* = 0.04), a Charlson’s comorbidity index ≥6 (*P* = 0.0001) and the development of ARDS (*P* = 0.04) significantly affected clinical outcome. Multivariate analysis showed that ARDS, Charlson’s comorbidity index ≥6 and treatment duration were independent predictors for overall clinical cure (*P* < 0.0001).

Overall, in-hospital patient mortality was 14 % [27/192] but mortality due to pneumonia was 9 % [17/192].

Where patients developed ARDS, in-hospital mortality was significantly higher (37 % [11/30] versus 10 % [16/162], respectively; *P* = 0.0005). Overall relative risk (RR) for mortality in the presence of ARDS was 3.7 (95 %, confidence interval (CI) 1.9–7.2). No difference in overall mortality and in mortality related to the episode of pneumonia was observed between groups, neither in patients hospitalised in ICU nor in those developing ARDS (see Table 2). Univariate analysis found that the development of ARDS, a Charlson’s comorbidity index ≥6 (*P* = 0.02) and treatment duration (*P* < 0.0001) affected mortality. Multivariate analysis found that these three variables were all independent predictors for in-hospital mortality (*P* < 0.0001). A sub-analysis of the impact of the length of treatment on the overall mortality showed that a treatment of 5 days or less is strongly associated (*P* < 0.0001) with an increased mortality (38 % [15/39]) when compared to longer treatment durations (8 % [12/153]). A trend for higher mortality is still observed when the treatment duration is of 7 days or less (16 % [25/155]) when compared to longer treatment durations (5 % [2/37]), although this difference does not reach statistical significance.

**Table 2:** Mortality rates per treatment group

Mortality	Piperacillin/tazobactam	Amoxicillin+temocillin	*P*-value
Overall mortality	15 % [14/94]	13 % [13/98]	NS
Hospitalised in ICU	44 % [8/18]	28 % [5/18]	NS
ARDS	38 % [6/16]	36 % [5/14]	NS
Mortality due to pneumonia	11 % [10/94]	7 % [7/98]	NS
Hospitalised in ICU	33 % [6/18]	11 % [2/18]	NS
ARDS	25 % [4/16]	14 % [2/14]	NS

*NS* not significant, *ICU* intensive care unit, *ARDS* acute respiratory distress syndrome

### Incidence of significant diarrhoea and *Clostridium difficile* infection

**Fig. 2:**
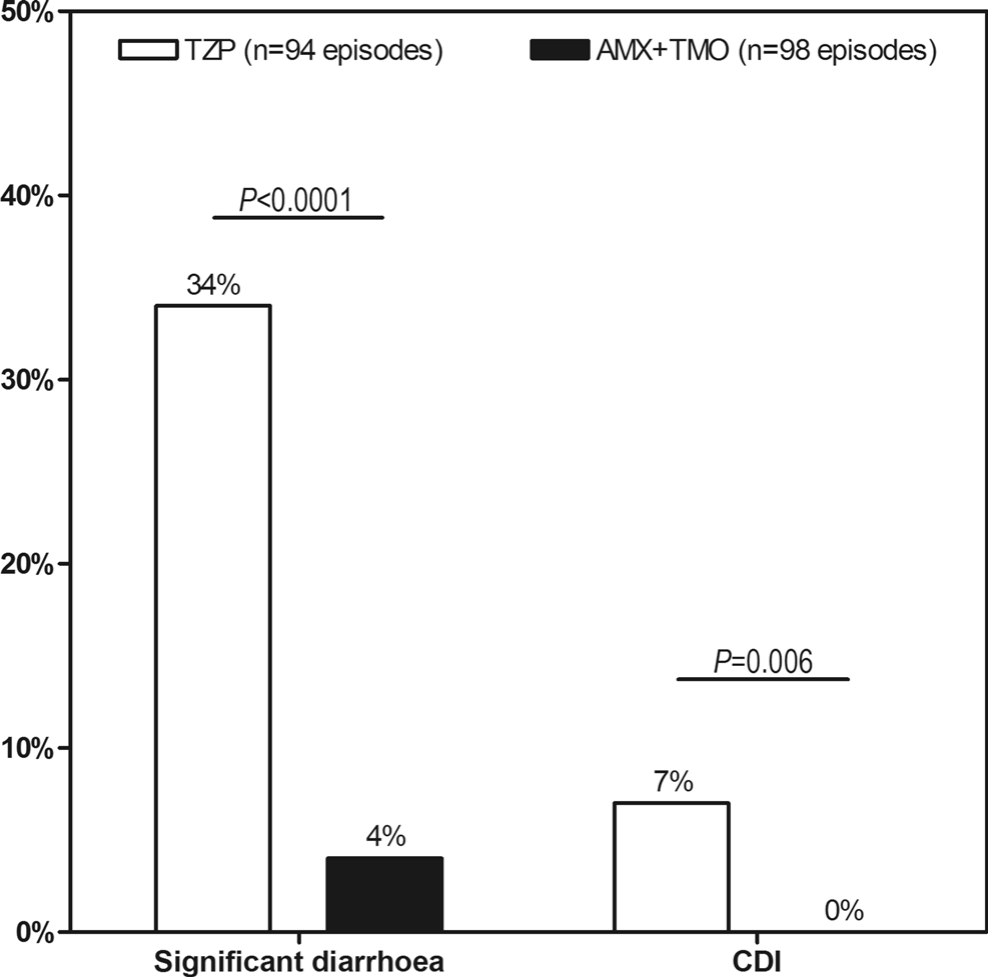
Adverse events of treatment groups. Frequency of significant diarrhoea and hospital-acquired *Clostridium difficile* infection (CDI) associated with piperacillin/tazobactam (TZP) [*white bar*] and amoxicillin plus temocillin (AMX+TMO) [*black bar*]. Significant differences were observed between groups

As shown in Fig. 2, in 34 % [32/94] of episodes treated with piperacillin/tazobactam, patients developed significant diarrhoea versus 4 % [4/98] for amoxicillin plus temocillin (*P* < 0.0001). This corresponded to a RR of developing severe diarrhoea associated with piperacillin/tazobactam treatment of 8.3 [95 % CI 3.1–22.7] when compared to amoxicillin plus temocillin.

The difference in the number of CDI cases between the two treatment groups also achieved statistical significance (7 % [7/94] of episodes for piperacillin/tazobactam versus 0 % [0/98] for amoxicillin plus temocillin, *P* = 0.006). No other factor affecting the incidence of CDI was identified on univariate analysis and, on multivariate analysis, the only independent predictor for CDI identified was the treatment group (*P* = 0.001).

Occurrence of diarrhoea increased hospital length of stay (LoS) (27 ± 2 days for episodes without diarrhoea versus 38 ± 3 days for episodes with diarrhoea, *P* = 0.005) and CDI further increased LoS (29 ± 1 days for episodes without CDI versus 49 ± 7 days for episodes with CDI, *P* = 0.0077).

## Discussion

A change in treatment from piperacillin/tazobactam to temocillin plus amoxicillin for HAP reduced the overall consumption of piperacillin/tazobactam by 30 % in our centre. The retrospective audit found no difference of clinical outcome between the two groups, although significant differences were observed in terms of gastrointestinal events. There were fewer cases of significant diarrhoea and no cases of CDI reported in patients managed with amoxicillin plus temocillin.

Combination therapy may offer an increased likelihood of adequate coverage for the potential pathogens causing the infection and a synergistic effect [13]. Those factors may be beneficial in terms of clinical outcome, for preventing and delaying the emergence of resistance and for shutting off toxin production [13]. Synergism with dual beta-lactam therapy may result from the enhanced inhibition of the penicillin-binding proteins (PBPs) and could offer better outcomes with less toxicity than beta-lactam-aminoglycoside combination regimens. Combination regimens including temocillin have been reported in in vitro studies where its association with compounds such as ampicillin, flucloxacillin and ticarcillin were synergistic or partly synergistic with an enhanced activity against some strains of *Pseudomonas* or *Staphylococcus* [14].

The rate of diarrhoea associated with piperacillin/tazobactam reported in the present audit is high but similar to the percentage of diarrhoea reported by Bow et al. [15]. This may reflect the demographics of the population which included a high proportion of elderly patients where an increased rate of adverse events associated with piperacillin/tazobactam has already been reported by Karino et al. [16].

There is a lack of consensus in the published literature relating to the incidence of gastrointestinal complications and the use of piperacillin/tazobactam. It has been reported that piperacillin/tazobactam inhibits growth and toxin production of *C. difficile* [17] and that during a shortage of piperacillin/tazobactam when replacement antibiotics were initiated, rates of CDI increased by 200 % or more in two institutions [18, 19]. However, the use of piperacillin/tazobactam has also been associated with CDI. In one institution, the incidence of CDI decreased by 47 % during the period in which there was a shortage of piperacillin/tazobactam [20]. Other studies have identified piperacillin/tazobactam as a risk factor for CDI [21–23]. In the present study, a CDI rate of 7 % was reported, a figure similar to other reports [24, 25]. Compared to cephalosporins and clindamycin, piperacillin/tazobactam is not a strong inducer of CDI; yet, a recent meta-analysis reported a 50 % increased risk of hospital-acquired CDI to be associated with penicillin combination antibiotics such as piperacillin/tazobactam [26]. In our centre, the use of piperacillin/tazobactam rose from 92 defined daily dosage (DDD) per month in 2008 to 267 DDD per month in 2011. This increased usage may account for the differences in diarrhoea and CDI in this audit. We have also observed that a change from branded piperacillin/tazobactam to a generic seems to be associated with an increase in side effects, although these data were not collected in the current audit.

The present study supports the use of alternatives to broad-spectrum antibiotics, not only because broad spectrum agents are associated with the development of multidrug-resistant bacteria, but also because of their association with CDI [27]. In this context, temocillin will provide an alternative choice as animal models have not reported any induction of antibiotic-associated colitis after temocillin treatment [28]. Human studies also suggest temocillin is safe for the individual patients with regards to ecological side effects [7, 29].

In our hospital in 2013 and 2012, two and six cases of CDI associated with the use of piperacillin/tazobactam were reported. These cases accounted for 20 and 42 % of all our hospital-acquired cases in 2013 and 2012, respectively, while in 2011 and 2010, the cases associated with piperacillin/tazobactam (17 and 23 cases, respectively) accounted for 71 and 62 % of all our hospital-acquired cases. This is consistent with the findings of a recent Cochrane review which highlighted that interventions to reduce excessive antibiotic prescribing to hospital inpatients can reduce antimicrobial resistance or hospital-acquired infections, and that interventions to increase effective prescribing can improve clinical outcome [30].

There are limitations to the use of the combination amoxicillin plus temocillin that need to be highlighted. This combination is only suitable in centres with a low incidence of *P. aeruginosa* (< than 5 %) as neither antibiotic is active against it. Moreover, it is important to include provisions for other organisms not covered by the combination (such as *S. aureus* and anaerobes) which is not appropriate empiric treatment for VAP.

Data collected in the present audit were recovered retrospectively. It is widely acknowledged that retrospective studies are exposed to bias, and that caution should be exercised in interpreting these findings. Only a limited number of patients included in the present audit had a positive bacterial culture. The diagnostic sensitivity of the sputum culture is known to be variable, between 29 and 94 % [31]. Such variable results may be related to inadequate sampling of sputum (difficulty of elderly patients to produce enough sputum), delayed processing of sputum specimens, and prior antimicrobial therapy (which occurs in 50 % of the patients in the present study) [31].

In conclusion, our audit suggests that amoxicillin plus temocillin is a viable therapeutic option in the treatment of severe HAP, and is associated with a favourable safety profile when compared to piperacillin/tazobactam. Further non-inferiority randomised controlled trials are needed to confirm those preliminary data, and to better define the place of amoxicillin plus temocillin as potential empirical treatment of severe HAP. Audits are necessary to validate formulary changes and sharing these data may encourage other centres to conduct randomised controlled trials using this or another combination of antibiotics in order to minimize the over-use of broad-spectrum antibiotics such as piperacillin/tazobactam and carbapenems.
